# Complex network models reveal correlations among network metrics, exercise intensity and role of body changes in the fatigue process

**DOI:** 10.1038/srep10489

**Published:** 2015-05-21

**Authors:** Vanessa Helena Pereira, Maria Carolina Traina Gama, Filipe Antônio Barros Sousa, Theodore Gyle Lewis, Claudio Alexandre Gobatto, Fúlvia Barros Manchado - Gobatto

**Affiliations:** 1 University of Campinas, School of Applied Sciences, Laboratory of Applied Sport Physiology, Limeira, SP, 13484-350, Brazil; 2 Naval Postgraduate School, Center for Homeland Defense and Security, (Emeritus) Monterey, CA, 93943, United States

## Abstract

The aims of the present study were analyze the fatigue process at distinct intensity efforts and to investigate its occurrence as interactions at distinct body changes during exercise, using complex network models. For this, participants were submitted to four different running intensities until exhaustion, accomplished in a non-motorized treadmill using a tethered system. The intensities were selected according to critical power model. Mechanical (force, peak power, mean power, velocity and work) and physiological related parameters (heart rate, blood lactate, time until peak blood lactate concentration (lactate time), lean mass, anaerobic and aerobic capacities) and IPAQ score were obtained during exercises and it was used to construction of four complex network models. Such models have both, theoretical and mathematical value, and enables us to perceive new insights that go beyond conventional analysis. From these, we ranked the influences of each node at the fatigue process. Our results shows that nodes, links and network metrics are sensibility according to increase of efforts intensities, been the velocity a key factor to exercise maintenance at models/intensities 1 and 2 (higher time efforts) and force and power at models 3 and 4, highlighting mechanical variables in the exhaustion occurrence and even training prescription applications.

## A New Model of Fatigue

The aim of this study is to understand the fatigue process in physical exercise and how it affects the entire environment of contracting muscles to generate force, power, heat and metabolic rates that affect the equilibrium of the internal environment, the associated generation of mechanical energy, and the feelings of fatigue^1^. The main physiological role of fatigue is to protect the body against traumatic effects of exercise^1^,^2^. Intensity, time, resistance, and type of exercise are variables that have different effects within bodily systems. How are exercise, fatigue, and complex networks related?

In the late XIX century, Mosso (1904) first suggested that fatigue at first glance seems like an imperfection of our body, but it is one of its most wonderful perfections^3^. How? When fatigue increases faster than the amount of effort, it minimizes the possibility of damage, thus its main function is protection. Both the brain and muscles change their functions during exercise, and fatigue is part of a complex control system or network, whose main goal is to protect the body from damage. These feelings of fatigue are unique to each individual, and the mental decisions made by winners and losers, both in training and in competition, are the ultimate determinants of performance^2^. This means that the best way to analyze fatigue is through a complex dynamic model. Such observations could be made based mainly in the area of investigation called complex networks or network science, which has been successfully applied to investigate different systems with complex interactions, including biological systems^4^,^5^.

The “new science of networks” is an emerging field with ancient roots in the use of graph theory in 1736 to resolve practical problems^5^,^6^. A network is a graph *G* *=* *{N, M, f},* where *N* *=* *{n*_*1*_*, n*_*2*_*,… n*_*k*_*}* nodes; *M* *=* *{m*_*1*_*, m*_*2*_*,… m*_*l*_*}* links, and the mapping function is *f: N x N*. Here, *f* is expressed as a connection matrix *C*, which defines the network topology. In this paper, nodes represent factors relating to fatigue, and links represent interactions among these factors.

Each model that we propose here can be represented as a network. Performance can also be limited by several physiological, biochemical, environmental and psychological factors^1^,^2^. A more realistic model could be an exercise-induced global complex model, including changes at distinct levels, at distinct intensities, in order to clarify interrelated factors and complex models closer to the real dynamic situation of fatigue occurrence. Others studies have tried to understand the sensation of fatigue in sedentary and trained individuals when subjected to a long, high-intensity exercise^7^,^8^ or high volume exercise^9^. There are physiological explanations related to the central nervous system^10^,^11^ and to mechanical failures linked to muscle capacity and individual energy^12^,^13^. However, none of these works have tried building and showing a model of interactions that represents the dynamic processes involved. Aimed to analyze this process, in the present study, we assume fatigue as non-maintenance of physical exercise assessed by individual time to exhaustion (or time limit–tlim) in different intensities.

Running in a non-motorized treadmill is a type of laboratory exercise used to acquire accurate values of power, with high reproducibility, that can be applied to the analysis of fatigue, muscle recruitment patterns, measurement of power, velocity and force, as well as correlations between these variables and kinematics^14^,^15^,^16^. This exercise model is efficient to measure variables such as mechanical power, considering the running specific gestures and not the ones on the cycle ergometer^17^,^18^. Besides, through the modern techniques of computer monitoring and signal capture, it becomes possible to determine the individual aerobic and anaerobic measurements^19^,^20^.

From the available literature, there are no reports of complex models being constructed to represent the variables measured in the exercise situations studied here. Therefore, this study is the first to combine network analysis with a critical power model to analyze exhaustion occurrence. Recently, the need for studies on the theory of complex networks to expand a new field, the physiological networks, has been proposed, since the topology of networks has been shown to be directly related to bodily/physiologic functions, across different sleep stages^21^. However, research studies focused on the exercise process to understand the development of fatigue as a complex network model, capable of considering different levels of exercise efforts, are not available in the literature. Thus, the main aim of the present study is to analyze the exhaustion at different intensity efforts and to investigate its occurrence as interactions at distinct body changes during exercise, using complex network models.

This is the first study to propose a complex network model of exercise performed at distinct intensities. We show that exercise is a dynamic process combining mechanical and physiological factors with theoretical and mathematical foundations. Changes at mechanical and physiological levels are represented as a complex network. Furthermore, the complex network model shows how these interaction patterns and the places of the nodes in the structure can reveal the role of each parameter/node in body changes.

## Results

### Mechanical and physiological variables involved in the fatigue process

**Figure 1:**
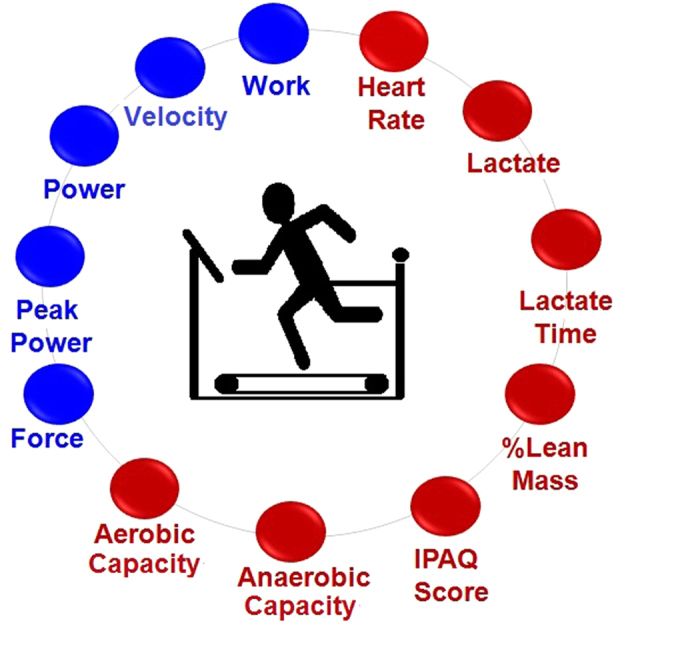
Proposed complex network model. The nodes are measurements of changes in body systems at the mechanical (blue) and physiological (red) related levels and IPAQ score (red) during four different intensities of exercise tests.

The proposed model has the measurements, calculations and format shown in Fig. 1. Connectivity (links) varies at four different exercise intensity levels (four tests). The parameters were separated into two groups: mechanical (force, peak power, mean power, velocity and work) and physiological related responses (heart rate, blood lactate, time until peak blood lactate concentration (lactate time), lean mass, anaerobic capacity and aerobic capacity) and IPAQ score.

Table 1 and 2 shows the mean values and standard deviations of the variables measured on the fatigue process, at four intensities in distinct scenarios, significances tests and comparisons, which revealed consistent differences between intensities. By developing a software program in Java language, we built four different complex networks based on influences. Also, using this program we calculated the following network properties and measurements: Degrees, Eigenvalues and Betweenness centrality of each node. Nodes and links are entered into the Java program as follows:

A node is a measurable attribute, as shown in Fig. 1. A link is an influence: node *A* is linked to node *B* if *A* is an influence on *B*, denoted *A* *→* *B*. The correlation coefficient of link *A* *→* *B* is a measure of the influence of node A on node B. Correlations were normalized by dividing them by the maximum correlation value over all links. Connection matrix *C:* matrix *N* × *N* of links connecting nodes: *C*_*(i, j)*_ *=* correlation result calculated between two measurements (nodes). *C* is symmetric when links are bidirectional, e. g. *i* ´ *j*. Then *C*_*(i, j)*_ *=* *C*_*(j, i).*_ If *C* is non-singular, its eigenvector *V* *=* *{v*_*1*_*, v*_*2*_*,…v*_*k*_*}* where *v*_*i*_ are eigenvalues corresponding with nodes *n*_*i*_. Then the solution to *[C-VI]* *=* *0*, where *I* is the identity matrix, yields the eigenvalues *V*. The degree of a node is the number of connecting links. The betweenness centrality of node *A* is the number of shortest paths passing through node *A* as determined by counting all the shortest paths from all nodes to all other nodes.

Links represent the presence of moderate or high level of correlations between nodes. These correlations are bidirectional, which means possibility of influence in both directions. Let influence vector *S*_*(0)*_ initially be defined as the initial state of nodes *N*. Then the next *state S*_*(1)*_ *=* *C* x *S*_*(0)*_*, S*_*(2)*_ *=* *C* x *C* x *S*_*(0)*_ *=* *C*^*2*^ x *S*_*(0)*_, etc. Therefore, *S*_*(t)*_ *=* *C*^*t*^ x *S*_*(0)*_. But, since *VI* can be substituted for *C*, because *[C-VI]* *=* *0; S*_*(t)*_ *=* *[VI]*^*t*^ x *S*_*(0).*_ Therefore, the state of node *i*, represented by *s_i_*, is asymptotic to *s*_*i*_ *=* *v*_*i*_^*t*^ x *s*_*i(0)*._ Obviously, *s*_i_ approaches infinity if *v*_*i*_ *>* *1*, and approaches zero if *v*_*i*_ *<* *1*. Nonetheless, *v*_*i*_ may be interpreted as the influence of node *n*_*i*_ on the network. Thus, *v*_*i*_ is a measure of the influence of *n*_*i*_ on, or importance to, the network^5^,^22^.

**Figure 2:**
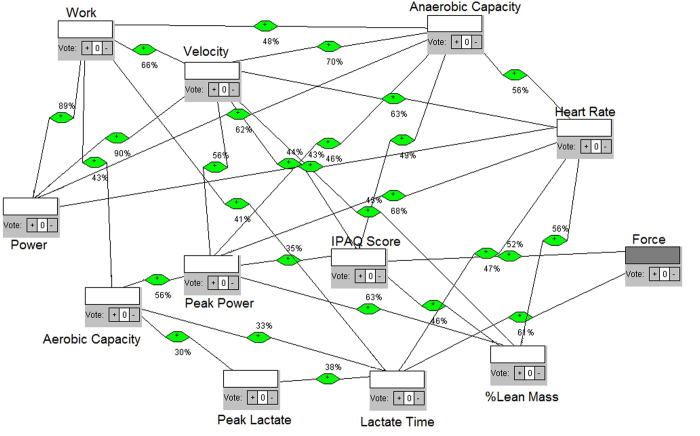
Proposed complex network model of influences, intensity/model 1.

**Figure 3:**
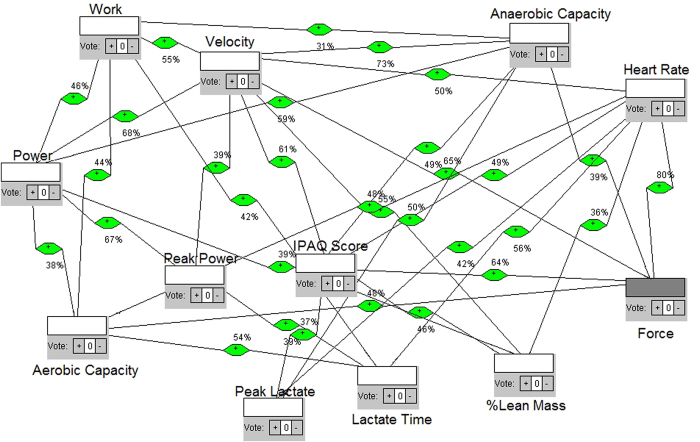
Proposed complex network model of influences, intensity/model 2.

**Figure 4:**
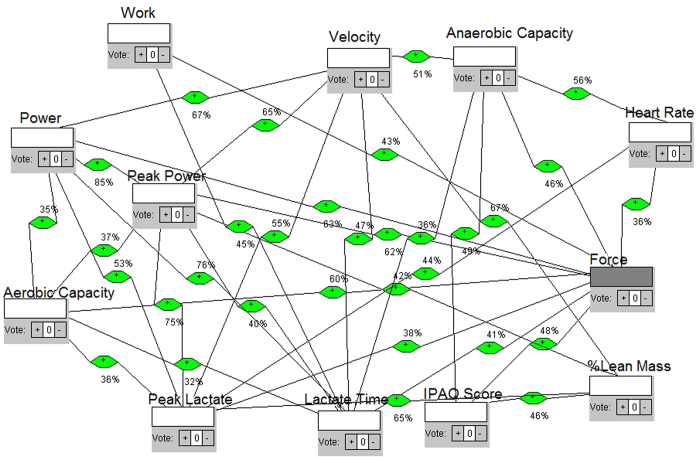
Proposed complex network model of influences, intensity/model 3.

**Figure 5:**
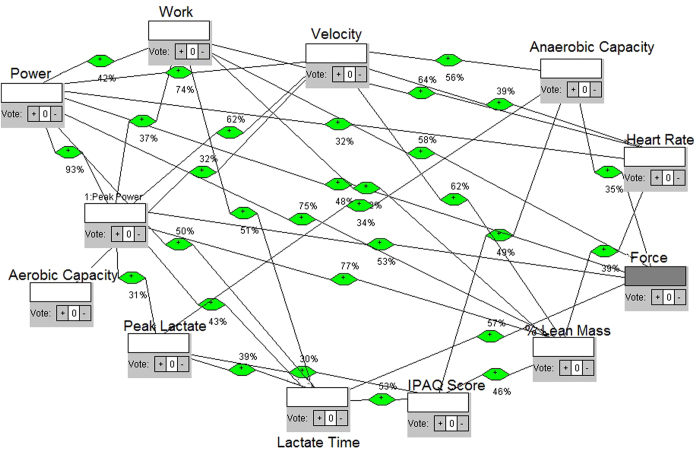
Proposed complex network model of influences, intensity/model 4.

We define each model as a network with different intensity of effort obtained from Table I. The four intensities and each test were differentiated according to elastic number (3, 4, 5 and 6 elastics for intensities 1, 2, 3 and 4, respectively), then the individuals must win theses resistances making force till exhaustion. Each intensity gave us feedback for next intensities. They were tethered and oriented to keep velocity and place in the treadmill, but force was determinant parameter of intensities. The four models are shown in the Figs. 2, 3, 4 and 5, respectively. Each one shows how fatigue evolves with effort intensity (1, 2, 3 and 4). The force was measured directly and used a high frequency of signals capture (1000 Hz). This parameter was capable of distinguishing intensities and depends only on the individual’s performance. All them keep the location in the treadmill and the position of the belt (between waist and hip). This allowed us to reproduce in the laboratory conditions similar to field tests, as in prior studies^23^,^24^,^25^.

In model 1, Fig. 2, along the 28 connections, the node that has more connections (hub node) is velocity; which means its influence over the other nodes is probabilistically greater. The mean time until exhaustion was 626 seconds, the greatest time comparing all 4 models, explained by the smaller effort expended. Analyzing the eigenvalues, which point to a possible convergent point of the entire system, velocity is also the greatest node. The higher betweenness centrality shows the flow behavior considering the structure of the network, and the node that shows the greatest number of paths was peak lactate time (lactate time), a physiologic-related measure. Model 2, Fig. 3, shows 31 connections, at 462 seconds of mean time limit. The hub, the maximum eigenvalue and the main betweenness centrality nodes were the same: velocity.

**Figure 6:**
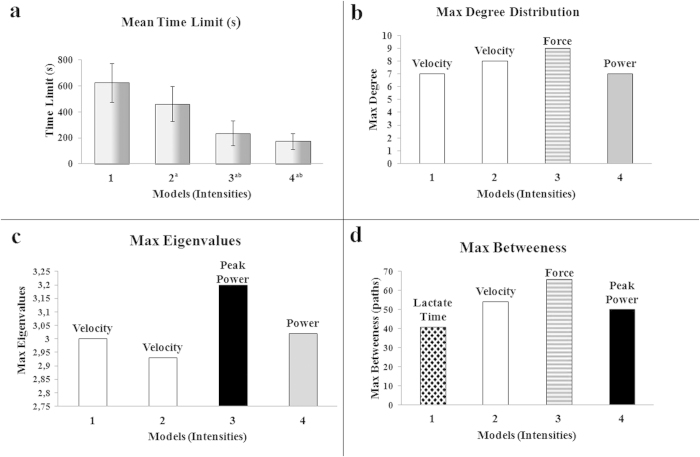
(**a**) Mean time limit and ±s.d. in which time until fatigue was inversely proportional to effort intensity, ^a^ Substantial difference between intensity 1, ^b^ intensity 2 , ^c^ intensity 3. (One-way ANOVA followed by Student-Newman-Keuls test, n = 9, P < 0.05). (**b**) Max degree distribution, hub nodes that are more correlated and therefore connected to others. (**c**) Max eigenvalues, at lower efforts (Models 1 and 2), velocity had major contributions; at higher efforts, force and power had greater value and contribution. (**d**) Max Betweenness, which considers the flow of information across nodes; the betweenness centrality was greater in model 3, with the main mechanical contribution; in model 4, Peak Power was the most influential. Max degree distribution: it appoints the major influent nodes, with the greater number of connections (hub nodes); Max Eigenvalue: the maximum eigenvalue of a network meaning the gravitational pull exerted by each node on the overall network. Higher eigenvalues mean more influence over other body systems. An eigenvalue greater than 1.0 means the network is unstable, though; an eigenvalue of zero means the node has no influence. The betweenness centrality: the amount of control exerted by links over the flow of information, expressed in terms of paths.

Model 3 in Fig. 4 shows a higher number of connections, 32. The node which has more connections (hub node) is now force, showing an increased contribution of the mechanical characteristics in the fatigue process. The mean time until reaching fatigue was 236 seconds, almost half as much time as the second model. The node with the maximum eigenvalue changed too; it is now Peak Power, showing its dependence and convergence point to time variations. The betweenness centrality node was force, that is, the flow of information and the probabilistic effects are the same variable. The fourth model, at Fig. 5, shows 30 connections, the hub node was Power and the mean time until fatigue was 173 seconds, almost a quarter as much time as the first model. In relation to eigenvalues, the higher one was Power, too. The main betweenness centrality node changed, and it is now Peak Power. The results are summarized in Fig. 6.

## Discussion

From the conception, development, construction, and analysis of each model based on each response obtained by four distinct effort intensity scenarios, we made the network metrics and observe that mechanical variables, performance-related to subjects (velocity, force and power), appear most commonly as hubs, i.e., these mechanical nodes are the most influential nodes. In our point of view, changes and variations in these specific mechanical responses have greater influence on other nodes/parameters, as they are more interrelated and connected with the entire network. Comparing models and analyzing lower intensities, velocity had the main probabilistic influence. Analyzing higher intensities, force and power probabilistically had the greatest influences over all network.

This findings confirms the reality and validity of the model. Such nodes, subdivided into mechanical, can be linked to muscular fatigue and are studied by other authors^12^,^13^,^26^. We know that peripheral information processing helps to reach high level of fatigue^27^. When we measure the eigenvalues and interpret the results, we can see that the variables that are directly or indirectly physiological, i. e., by analyzing the tendency of convergence of the model, there is a dependency on physiological-related variables (e.g. lactate time), notably when effort is smaller. When analyzing an increase in amount of effort, we observe that the time until fatigue gets shorter, and the dependency is focused on the nodes directly connected to the main one responsible for muscle strength generation.

Because of these observations, we can say that peak lactate time as another physiologically important variable, that should be considered in fatigue analysis. Smaller effort over a longer time can make the flow of information between body systems pass through changes in variables related to time and displacement, mainly velocity, which is reflected in maximum degrees and eigenvalues in models 1 and 2. Moreover, when we analyze greater effort, the flow information seems to pass by force and power. This leads us to point out that the dependence of the oscillations is also related to time and an evolutionary dependence on the mechanical variables (force and power) of the systems. This observation reinforces the idea of a complex dynamic system of interactions that changes the fatigue behavior, not just the idea of less force, longer time until fatigue^28^ or fatigue specifically related to the strategy adopted by individuals^29^.

Our idealized models are based on the existence of correlations between the chosen and measured variables. Correlations were used to quantify the connection between nodes (variables). Low correlation indicate low or non-existent contribution of participation. High correlation indicated strong contribution or participation. Correlations were shown for several network models to demonstrate the relevance of the network analysis. This approach is similar to the approach of others network analysis^21^,^30^,^31^ where the emphasis was on parameters of greatest contributions.

Explanations of these key contributions can also be related to Physics. Velocity is a measure that involves displacement under time. In the first two models, velocity is highlighting itself. Thus, what matters the most at smaller efforts and longer time is the displacement. Force involves mass and acceleration. In model 3, force is highlighting itself. Acceleration involves velocity variation under time. When efforts start to be greater, under time reduction, velocity variation is more important.

In model 4, power is the more relevant node. Power is a result of work under time. Work is a result of force and displacement under time. Force involves mass and acceleration, which involves velocity variation^32^. In other words: *P* *=* *W /T*, where *P* is power, *W* is work and *T* is time. *W* *=* *S.D*, where *S* is force and *D* is displacement. Then, *P* *=* *(S.D)/T*. But, *S* = *ma*, where *m* is mass and *a* is acceleration. Then, *P* *=* *(maD)/T*. *a* *=* *v/T* where *v* is velocity variation and *T* is time variation. Therefore, *P* *=* *(mvD)/(TT).* Thus, we have *P* *=* *Sv*. Thus, the evolution of effort (effort becoming greater and time becoming shorter) involves more mechanical variables (power as a result of force and velocity). In the first models, force and velocity seem to show their importance singly and separately. Looking at all this, these models show consistency, because Physics facts can be confirmed by the network metrics and other works.

Considering the applicability of our results in training prescription, when the goal is increase performance in long runs, the focus should be on individuals training to improve velocity. It is a fact that running distances of 3000 m are commonly performed in velocities above the aerobic capacity^33^. That way, runners specialized in such distances should practice to improve the velocity which they could tolerate in 7 to 10 minutes – time durations similar to model 1 and 2. Also, results of the present investigation indicate that at greater effort and shorter time, force and power are the main protagonists. Thus, at running distances that would last 2 to 4 minutes (800 m to 1500 m), athletes should focus their training on improving force and power. This hypothesis is in accordance with the studies linking resisted training to performance improvement in shorter running distances^34^. Such variables and their relationships, specially force and velocity, also has been investigated and demonstrated in exercise^35^,^36^ and our network metrics results reinforces such previous results, including the importance of studying parameters across a range of distinct intensities. All these interpretations and models proposed in this work show great promise as a new framework for exercise physiology analysis. These findings agree with the sports sciences evidence so far.

We can see that changes happen according to time and effort intensity, but in a new way of analysis and in a richer range of interpretations, not seen before, with larger contribution variables over a range of exercise intensities. Moreover, the time until peak blood lactate concentration (lactate time) is important and contributes positively to the fatigue models. In most cases, different from what was done here, blood lactate is isolated analyzed^37^ but, interestingly, the network approach did not show blood lactate as a key factor for fatigue, similarly to other studies^38^ Also, we analyzed time to reach peak lactate, and show that it is a convergent point of the system.

These models can be applied to analyze performance in other types of exercises to better manage time fatigue in athletes from different sports. The network metrics show sensibility in parameters behavior over a range of intensities. These findings extend the analysis of fatigue that considers specific causes or even no communication with various bodily systems, changes and reveals the need to build complex models that allow the study of an entire environment of changes to understand the ways of exhaustion occurs. Here, we did not study a small section of the changes to obtain the answers to what happens in the whole body. Instead, we are showing the need to better represent and understand the global context of changes in which fatigue occurs.

In summary, our results suggested the interesting complex network analysis to study different exercise intensities. Additionally, the nodes, connections and network metrics show sensibility according to efforts intensities, been the velocity a key factor to exercise maintenance at models/intensities 1 and 2 and force and power at models 3 and 4. This work therefore provides the potential emergence of a new approach: body systems/changes represented by measurable parameters and responses that can show a larger complex network that better represents what is happening in the whole body during many types of activities in our daily lives.

## Methods

Nine individuals (mean age, weight, height and fat percentage of 24 ± 4 years, 78.5 ± 9.1 kg, 179 ± 8 cm, 9 ± 3.5%, respectively) ran on a non-motorized treadmill tied by elastic cable (tethered running) were analyzed at four intensities. This study was approved by the Research Ethics Committee of University of Campinas, School of Medical Sciences (protocol no. 07716512.1.0000.5404), in accordance with the Declaration of Helsinki. Verbal and written informed consent was obtained from all participants, which reported physical activity at least three times a week. Also, they signed a free and informed consent form, that contains information about procedures, confirming voluntary participation and consent to the use of data for further scientific publications, and certify the non-use of any illegal substances. The participants were instructed to keep a light diet and well hydrated and perform the last meal at least two hours before tests, do not consume beverages containing alcohol at least 24 hours before tests and caffeine at least 4 hours before tests; besides they could not practice strenuous exercises during the experimental period. All tests were executed in a laboratory environment. The mean temperature was 23 °C (controlled by air conditioning) and relative humidity ranged from 30 to 40% (Thermo hygro decibelimeter lux digital multimeter, THDL 400, Instrutherm). Six visits at laboratory all necessary for data collection and a minimum interval of 24 hours among tests was observed. In the first day, the anthropometric assessment and adaptation to ergometer were carried out. Values of height, weight (scale model Toledo® 2098 column 1.0 m) and body fat (skinfold scientific Sanny®) were obtained used a specific protocol^39^. The individual adaptation process consisted to 30 s races at different velocities, varying of mild, moderate, intense and very intense. After this adjustment, four days were used for the application of standard protocol to determine the critical power model parameters. Before each test, all participants were submitted to warm up for 5 minutes composed by a running at 7.0 km/h, using the motorized treadmill (Model Super ATL, Inbramed, Brazil). Volunteers ran on a non-motorized treadmill tethered by a steel cable attached to an elastic system. The resistance imposed by the increase of the elastic force from the elastic in the system number (3, 4, 5 and 6 elastics differentiated intensities 1, 2, 3 and 4, respectively) and measured by a force pickup signal system. The duration of tests were planned considering the critical power model, that foresee aerobic and anaerobic determination from the relationship between intensity and time to achieve the exhaustion (time limit). We fixed a zone of 2 to 10 min^40^,^41^,^42^ for four intensities and each intensities test were differentiated according to elastic number, them the individual must win theses resistances making force until exhaustion. Each intensity gave us feedback for next intensities. However, we not fixed the velocities. They were tethered and oriented to keep velocity and place in the treadmill, but force was determinant parameter of intensities. The exhaustion criteria were defined by an apparatus developed by our research group. This apparatus was capable to inform both the participant and the evaluator about the maintenance of exercise. For example, when the subject did not sustain the initial position, the equipment fired a beep and, if they were not able to restore the necessary force to maintain the target position for more than five seconds, the time limit was reached. Additionally, the participants received constant verbal encouragement. Mechanical measures (force, velocity, power, work) were captured via signals (LabView Signal Express 2009 National Instruments®), by a hall-effect sensor with 1000 Hz acquisition, calibrated before each test, modulated (USB-6008, National Instruments®) and subsequently transferred to MatLab (R2008a MatLab®, MathWorkstm), using specific technical details avaiable^43^.

Aerobic and anaerobic capacities were obtained by hyperbolic critical power model: a hyperbolic relationship between power output and the time that the power output can be sustained; the power asymptote of the relationship, CP (critical power), can be sustained without fatigue. Time to exhaustion can be predicted for any power output ≥ CP from the hyperbolic relationship that includes anaerobic work capacity (AWC): *T*_*lim*_ *=* *AWC/ (P–CP).*^44^,^45^. To determine the blood lactate responses, 25 *μL* blood samples were collected from the earlobe using a heparinized capillaries, in five moments (rest, after five minutes of warming, after exhaustion, and 5^th^ and 8^th^ minutes after exhaustion), maintained in Eppendorf tubes (400 *μL* of trichloroacetic acid at 4%, 2 to 8 °C), determined at 340 nm (calibration of 5, 10, 15 and 30 *mM*, Engel & Jones, 1978). Peak lactate values were determined at a specific time for each individual, originating peak lactate time. The heart rate was recorded at rest and after test (Polar® monitor RS800CX). To build the models, each variable measured was represented as a node of the network (*mechanical:* peak power, velocity, force, work and power; *physiological:* heart rate, blood lactate, peak lactate time, aerobic capacity, anaerobic capacity and lean mass) and IPAQ score. To estimate the physical activity level of the participants, IPAQ instrument was used. This instrument corresponds to a compendium of physical activity^46^ converted into metabolic equivalents/min/week. According to characteristics of this questioner, it was applied only in a one moment of experimental period (first visit to laboratory).

For construction of the models, first we considered not only the proposition, but the theoretical and mathematical foundation to build the networks. We considered a measurement of the maximum number of variables that could reveal the occurrence of changes in various bodily systems in order to consider them as quantified node in a network. Then, we studied the best way to interrelate, i.e. to connect, each node. We then considered the calculation of correlations between sets of data available for each variable/node measured, by Pearson correlation. Whenever the result of the calculated correlation was considered as moderate or high, we set up the connection/link between such nodes. For this purpose, an algorithm was built with classes and functions in Java language, which received as vector data sets in a main function, passing them to the function built correlation, performing the calculations of correlations, from the set of data collected from each variable in each exercise intensity. After obtaining the values, a third function was constructed to check the type of correlation in relation to the range of values found. If the result was less than 0.3 or −0.3, correlation was considered weak, if greater than 0.3 and less than 0.7 or less than −0.3 and greater than −0.7, it was considered moderate, and when less than − 0.7 or greater than 0.7, it was considered high. The results were then displayed on screen, with double precision and printed with the intensity of effort: we showed the variables tested, the value found and the type of correlation observed. After such calculations and checks, high and moderate correlations were selected to establish connections and we noted their values for inclusion in the networks. Using the network analysis software, we then built the models.

Nodes were included and appointed by the addition class. All nodes were defined as neutral influences on the network, because the idea was to observe the behavior without defining it or to list positive or negative importance in the fatigue dynamics. Then, the links based on the values obtained by the Java functions and resulting correlations were inserted. Each link is also weighted with the result of the correlation value: for example, if the correlation had a score of 0.75, such link was weighted at 75% influence. Moreover, as the Pearson correlation shows the same result for both directions, we performed the suitability of the software for the weighted network that had all bidirectional links, i.e., exercised influence in both directions; since one node and the other one have no relation of cause and effect but are correlated, the result was a two-way weighted network. This weight was defined because, at most real-world networks, not all links show the same capacity. Actually, links are associated with weights that are different in terms of their intensity, capacity or flow^47^. This means that the models try to be as close as possible of what is really happening, mathematically grounded. After the networks were built, the simulation was run to calculate the degree of the nodes and then displayed on screen. The program displays the results with the degree of each node and points to the node with the highest degree, i.e. the higher number of connections (hub node) which therefore have greater energy of influence. After that, there were calculations functions for eigenvalues of nodes. The program calculates every node eigenvalue and shows the eigenvalues of each node, highlighting the node of maximum eigenvalue. This measure helps the understanding of nodes influence, such as other research in complex systems^48^. Furthermore, there was a simulation of the betweenness centrality of the nodes, in which the system calculates and shows the betweenness centrality of each node and points to the node of greatest betweenness centrality, indicating the proximity of this in relation to the other nodes. Each network construction, simulation and calculation was made for each model representing a different exercise intensity.

## Author Contributions

V.H.P., T.G.L., F.B.M.G. and C.A.G. propose models ideas, wrote the main manuscript text, interpreted data, created the models and prepared figures. M.C.T.G. and F.A.B.S. collected data from laboratory tests and analyzed it. T.G.L. developed the network software. V.H.P. and T.G.L. design and build complex models and made metrics. All authors reviewed the manuscript.

## Additional Information

**How to cite this article**: Pereira, V.H. *et al.* Complex network models reveal correlations among network metrics, exercise intensity and role of body changes in the fatigue process. *Sci. Rep.* **5**, 10489; doi: 10.1038/srep10489 (2015).

## Figures and Tables

**Table 1:** Mean and ± standard deviations of parameters at each test intensity (1, 2, 3, 4).

**MEAN PARAMETERS VALUES AT EXERCISE INTENSITIES**				
	**1**	**2**	**3**	**4**
**Power (W)**	247.84 ± 47.46	294.46 ± 40.18	412.71 ± 93.75^ab^	512.42 ± 105.25abc
**Peak Power (W)**	380.34 ± 48.91	445.23 ± 48.79	585.19 ± 123.14^ab^	784.63 ± 96.00abc
**Force (N)**	120.05 ± 11.32	136.65 ± 17.53	161.86 ± 25.30^ab^	187.90 ± 27.55abc
**Velocity (m/s)**	2.11 ± 0.33	2.19 ± 0.38	2.54 ± 0.40	2.77 ± 0.56^ab^
**Time limit (s)**	626.08 ± 149.27	462.67 ± 133.29^a^	236.64 ± 97.24^ab^	173.75 ± 62.68^ab^
**Work (kJ)**	162.45 ± 56.04	135.48 ± 45.02	92.00 ± 25.03^ab^	77.73 ± 16.15^ab^
**Peak Lactate (mmol/L)**	12.20 ± 3.38	12.52 ± 4.49	15.12 ± 5.00	15.34 ± 5.49
**Lactate Time (s)**	819.41 ± 287.44	709.33 ± 260.12	583.30 ± 219.01	433.75 ± 251.68^a^
**Heart Rate (bpm)**	180.11 ± 10.54	179.00 ± 9.82	180.00 ± 9.53	179.55 ± 8.42

^a^ Substantial difference between intensity 1, ^b^ intensity 2 , ^c^ intensity 3. (One-way ANOVA followed by Student-Newman-Keuls test, n = 9, P < 0.05).

**Table 2:** Mean and standards deviations that characterize sample and became nodes to watch behavior in models dynamics over intensities.

**MEAN CALCULATIONS FROM SAMPLE**			
**Aerobic capacity (W)**	**Anaerobic capacity (kJ)**	**Lean mass (%)**	**IPAQ score (a.u.)**
139.26±	47.14±	91.49±	2106.66±
43.65	26.06	3.24	1162.06
